# Publication Audit - a useful tool to evaluate progress and plan for the future

**DOI:** 10.12669/pjms.312.7682

**Published:** 2015

**Authors:** Shaukat Ali Jawaid

Publication audit is considered a useful tool to evaluate the progress and plan for the future and we have been doing this exercise for the last couple of years.^1^-^3^ A careful look at the statistics for the Year 2014 reveals that there has been an increase in the number of submissions, a trend seen for the last few years consecutively. During the Year 2014, 1577 manuscripts were received from different countries. Table-I. A vast majority of the submissions were from China 524 followed by Turkey 441, Pakistan 255, Islamic Republic of Iran 107 and Kingdom of Saudi Arabia 88.

**Table I:** Country wise submissions during 2014.

Country	Total
Algeria	1
Australia	1
Bangladesh	8
Brazil	1
Canada	1
China	524
Egypt	2
Ethiopia	1
Fiji	1
France	3
Germany	1
India	24
Indonesia	2
Iran	107
Iraq	6
Italy	1
Jordan	2
Korea	14
Malaysia	37
Nepal	1
Nigeria	17
Oman	2
Pakistan	255
Palestine	1
Poland	3
Romania	10
Russia	1
Saudi Arabia	88
Serbia	1
South Arica	4
Sweden	1
Taiwan	2
Tunisia	1
Turkey	441
UAE	2
UK	1
USA	9
Total	1577

As regards submissions from Pakistan as expected most of the submissions were from Karachi 97 Lahore 40, Hyderabad and Peshawar 21 each while submissions from Islamabad and Rawalpindi were 18 and 17 respectively. Table-II. A total of three hundred one manuscripts were published during 2014 (acceptance rate of 19.8%) ten were rejected because of plagiarism while thirty manuscripts were withdrawn by the authors for various reasons. Table-III.

**Table II:** City wise submissions from Pakistan during 2014.

City	Total
Abbottabad	4
Azad Kashmir	1
Bahawalpur	5
Faisalabad	5
Gujrat	2
Hyderabad	25
Islamabad	18
	
Karachi	97
Khuzdar	1
Lahore	40
Malakand	1
Manshera	1
Mirpurkhas	5
Multan	7
Nawabshah	1
Peshawar	21
Quetta	1
Rawalpindi	17
Sawat	1
Sialkot	1
Toba Tek Singh	1
Total	255

**Table III:** PJMS manuscripts statistics of 2014 at a Glance

Total Articles Published:	301
Total Articles Rejected:	1192
Rejected because of plagiarism:	10
Articles withdrawn by authors:	30
Under process:	44
Total Articles Received:	1577

A vast majority of the published manuscripts during 2014 included original articles 248 followed by case reports 20 and Review articles ten. Table-IV. Among the published manuscripts ninety three were from Pakistan, eighty nine from China, sixty from Turkey and nineteen from Saudi Arabia. Table-V.

**Table IV:** Category Wise Manuscript Published in 2014.

Category	Jan-Feb 2014	Mar-Apr 2014	May-Jun 2014	Jul-Aug 2014	Sep-Oct 2014	Nov-Dec 2014	Total
Original Article	41	39	36	44	42	46	248
Case Report	1	5	4	4	3	3	20
Clinical Case Series	–	–	1	–	2	–	3
Short Comm.	–	–	3	1	–	1	5
Special Comm.	–	–	1	1	2	–	4
Editorial	1	–	–	–	–	1	2
Correspondence	2	–	1	–	–	1	4
Review Article	4	3	1	1	–	1	10
Conference Proceeding	–	–	–	–	1	–	1
View Point	–	–	1	1	1	–	3
Leading Article	–	–	1	–	–	–	1
Total	49	47	49	52	51	53	301

**Table V:** Country Wise Manuscript Published in 2014.

Country	Jan-Feb 2014	Mar-Apr 2014	May-Jun 2014	Jul-Aug 2014	Sep-Oct 2014	Nov-Dec 2014	Total
Pakistan	19	18	13	15	11	17	93
Iran	–	3	2	1	5	3	14
Turkey	12	8	13	8	9	10	60
China	14	12	13	18	18	14	89
Saudi Arabia	2	2	2	6	3	4	19
Romania	–	–	1	–	–	–	1
Malaysia	1	2	–	1	2	1	7
Bangladesh	–	–	–	–	1	–	1
Iraq	–	–	–	–	1	–	1
South Africa	–	–	–	1	–	1	2
USA	1	–	2	1	–	–	4
Korea	–	1	1	–	–	–	2
Palestine	–	–	–	–	–	1	1
Bahrain	–	–	1	–	–	–	1
Poland	–	–	–	1	–	1	2
Nigeria	–	1	1	–	1	1	4
Total	49	47	49	52	51	53	301

The number of submissions increased to 1577 in 2014 from 1023 in 2012 and 1091 in the Year 2013. Most of the submissions during 2012 and 2013 were also from Pakistan, Iran, Turkey and China and this has seen a continuous increase in the number of submissions from these countries over the years. It not only shows the popularity of the Pakistan Journal of Medical Sciences in these countries but also reflects that the authors are also satisfied with the processing and peer review system practiced by us. The biggest increase in submissions was witnessed from Peoples Republic of China starting from 189 in the Year 2012 to 325 in 2013 and 524 in the Year 2014. However, after pee review only 34 manuscripts from China were published during the Year 2012 and it increased to eighty in 2013 and eighty nine in the Year 2014. According to reports, China is the second biggest contributor to medical literature after United States of America which shows the tremendous research work being done by the Chinese research scientists and the recognition of research accomplishments by the Chinese Government. Similarly the number of manuscripts published from Turkey increased from thirty seven in 2012 to thirty eight in 2013 and eighty nine in 2014. The number of manuscripts accepted for publication after peer review from Saudi Arabia has also increased from sixteen in 2012 to seventeen in 2013 to nineteen in 2014. Kingdom of Saudi Arabia has also made lot of investment in the health sector and has established many new medical schools and tertiary healthcare facilities and the researcher’s contribution to medical literature is recognized and appreciated while making selection and academic promotions which has resulted in promotion of research culture in the Kingdom.

However, the number of submissions and manuscripts published after peer review from Islamic Republic of Iran has been constantly decreasing over the years. The number of published manuscripts from Iran in 2012 was sixty three which slightly increased in 2013 to seventy but decreased in the Year 2014 to just fourteen. It is because of the fact that now we are very selective and the authors from Iran are encouraged to publish their research work in their own biomedical journals. The number of medical journals published from Iran has increased from ninety in 2004 to 437 in 2014. Hence, the authors from Iran also find it more convenient to publish their research work in their own journals while it also saves them the publication charges since most of the journals published from Iran are run and managed by the medical universities and most of them do not charge any publication fee.

In view of the past experience and the reported developments we are now also very selective as regards accepting case reports, Reviews and Meta Analysis. Only very rare case reports are accepted while Reviews and Meta analysis also remains a very low priority with us and the authors are advised to get it published in their own country. Another reason for decreased submissions and publication of manuscripts from Iran is that many of the manuscripts which include KAP studies, Surveys, routine me too studies or those which just have local relevance are not accepted for further processing and the authors are advised to submit it to their local journals.

On the whole, over the years we are quite satisfied with the progress which we have made and now as per Scopus the well known international database, Pakistan Journal of Medical Sciences is included in the top Ten Biomedical Journals published from the Eastern Mediterranean Region. The other three medical journals from Pakistan included in this list are Journal of College of Physicians and Surgeons Pakistan (JCPSP), Journal of Pakistan Medical Association (JPMA) and Journal of Ayub Medical College, Abbottabad. (JAMC). Table-VI. While the other three Pakistani medical journals included in this list has backing and are affiliated with leading medical institutions and organizations, inclusion of Pakistan Journal of Medical Sciences in this list is a significant achievement considering the fact that it is a humble individual private effort. We are determined to further improve the quality of manuscripts accepted for publication, enhance the standard of the journal while every effort will also be made to reduce the processing and publication time remaining within our limited financial and human resources. However, at times the law and order situation in Karachi does affect the working which is something beyond our control. As the law and order situation improves, the processing and peer review process will be accelerated so that the authors do not have to wait for too long as regards processing of their manuscripts.

**Table-VI:** Top 10 Medical Journals published from the Eastern Mediterranean Region

No.	Journal Title	Country	H-index	Citation/paper	SJR
1	Saudi Medical Journal	Saudia	30	0.66	0.269
2	Archives of Iranian Medicine	Iran	25	1.25	0.369
3	Journal of Pakistan medical association	Pakistan	25	0.51	0.220
4	Journal of college of Physicians & Surgeons Pakistan	Pakistan	21	0.48	0.218
5	Current Aging Science	UAE	18	2.34	0.882
6	Pakistan Journal of Nutrition	Pakistan	17	0.37	0.249
7	Egyptian Society of Parasitology	Egypt	17	0.38	0.205
8	Journal of Ayub Medical College	Pakistan	16	0.11	0.161
9	Anti-Infective Agents	UAE	16	0.59	0.190
10	Pakistan Journal of Medical Sciences	Pakistan	15	0.14	0.134

Source: SCOPUS.
